# Canadian Initiatives to Prevent Hypertension by Reducing Dietary Sodium

**DOI:** 10.3390/nu3080756

**Published:** 2011-08-16

**Authors:** Norm R. C. Campbell, Kevin J. Willis, Mary L’Abbe, Robert Strang, Eric Young

**Affiliations:** 1 Departments of Medicine, Community Health Sciences, Pharmacology and Therapeutics, and Libin Cardiovascular Institute, University of Calgary, Calgary, Alberta T2N 4Z6, Canada; 2 Canadian Stroke Network, 600 Peter Morand Crescent, Ottawa, Ontario K1G 5Z3, Canada; Email: kevin@canadianstrokenetwork.ca; 3 Department of Nutritional Sciences, University of Toronto, Toronto, Ontario M5S 3E2, Canada; Email: mary.labbe@utoronto.ca; 4 Department of Health and Wellness, Nova Scotia PO Box 488, Halifax, Nova Scotia B3J 2R7, Canada; Email: Robert.Strang@gov.ns.ca; 5 Ministry of Health, 1515 Blanshard St., Victoria, British Columbia V8W 3C8, Canada; Email: Eric.Young@gov.bc.ca

**Keywords:** sodium, salt, hypertension, public health, nutrition, cardiovascular disease

## Abstract

Hypertension is the leading risk for premature death in the world. High dietary sodium is an important contributor to increased blood pressure and is strongly associated with other important diseases (e.g., gastric cancer, calcium containing kidney stones, osteoporosis, asthma and obesity). The average dietary sodium intake in Canada is approximately 3400 mg/day. It is estimated that 30% of hypertension, more than 10% of cardiovascular events and 1.4 billion dollars/year in health care expenses are caused by this high level of intake in Canada. Since 2006, Canada has had a focused and evolving effort to reduce dietary sodium based on actions from Non Governmental Organizations (NGO), and Federal and Provincial/Territorial Government actions. NGOs initiated Canadian sodium reduction programs by developing a policy statement outlining the health issue and calling for governmental, NGO and industry action, developing and disseminating an extensive health care professional education program including resources for patient education, developing a public awareness campaign through extensive media releases and publications in the lay press. The Federal Government responded by striking a Intersectoral Sodium Work Group to develop recommendations on how to implement Canada’s dietary reference intake values for dietary sodium and by developing timelines and targets for foods to be reduced in sodium, assessing key research gaps with funding for targeted dietary sodium based research, developing plans for public education and for conducting evaluation of the program to reduce dietary sodium. While food regulation is a Federal Government responsibility Provincial and Territorial governments indicated reducing dietary sodium needed to be a priority. Federal and Provincial Ministers of Health have endorsed a target to reduce the average consumption of sodium to 2300 mg/day by 2016 and the Deputy Ministers of Health have tasked a joint committee to review the recommendations of the Sodium Work Group and report back to them.

## 1. Introduction

Our distant ancestors consumed a low-sodium, high-potassium diet [1] and accordingly our kidneys are adapted to conserve sodium and excrete potassium [2]. We have known for over 2000 years that an acute high dietary sodium intake in the form of a salty [3] meal, results in a temporary increase in blood pressure and is associated with several other important diseases [4]. In developed countries, contemporary diets are high in sodium, primarily resulting from the salt added to manufactured foods and low in good sources of potassium such as vegetables and fruit. Our relatively recent chronic exposure to excessive dietary sodium intake is directly related to the development of high blood pressure [3,5], the leading attributable risk factor globally for non-communicable disease mortality [6]. Compelling evidence for excess sodium consumption being a major cause of high blood pressure includes data from primate studies [7,8], clinical trials [9], and human genetic studies [10,11]. The molecular mechanisms of how high sodium intake increases blood pressure are being elucidated [12,13].

The average sodium consumption in Canada among adults is approximately 3400 mg per day [14], over double the adequate intake of 1200 to 1500 mg per day depending on age recommended by the Institute of Medicine [15]. Recent surveys of the Canadian population finds 19% of adults has, or is being treated for hypertension (blood pressure higher than or equal to 140/90 mmHg) and a further 20% have pre-hypertension (blood pressure 120–139/80–89 mmHg) [16]. Recognizing that many developed and developing countries share this scenario and that reduction in the dietary sodium intake would have a significant impact on hypertension and cardiovascular disease, the World Health Organization has called for food companies to lower the sodium content of their products and for governments to introduce regulatory approaches if recommended sodium intake levels are not achieved through voluntary action [17].

## 2. Canadian Non Governmental Organizations (NGOs) Activities to Reduce Dietary Sodium

In 2006, the Canadian Institute for Health Research, the Canadian Hypertension Society and Sanofi Aventis funded a new leadership position—The Canada Chair in Hypertension Prevention and Control. Dr. Norm Campbell was appointed as the inaugural Chair. Canadian policy had called for reduced dietary sodium for several decades but Dr. Campbell recognized that a lack of a concerted health sector support might have hindered effective policy implementation. Leading NGOs to advocate for reducing dietary sodium in Canada, conducting research on the health impact of high dietary sodium and to educate health care professionals and the public about dietary sodium was one of the stated goals for the “Hypertension Chair” [18]. Blood Pressure Canada, a coalition of 27 national organizations agreed to adopt and support the mandate of the Hypertension Chair to reduce dietary sodium and at this time the Canadian Stroke Network (CSN) had also identified sodium reduction as the priority for stroke prevention in Canada resulting in strong coordinated national health care NGO support.

Initial NGO efforts were directed towards introducing guidance on reducing sodium into Canada’s Food Guide to Healthy Eating, which was being revised in 2006. A lobby effort involving ten major national health care and scientific organizations was organized to conduct a writing campaign to politicians and senior government officials as well as face-to-face meetings and attendance at public forums on the food guide was organized. The food guide, the major source of healthy eating information for Canadians was subsequently revised to include dietary sodium as a health issue of equal importance to sugar and saturated fats [19].

The next step was to develop a strategic planning committee with representation from eight major national health organizations to advocate for NGO, government and food industry action on dietary sodium. A policy statement was developed for Blood Pressure Canada by the strategic planning committee calling on the Canadian government and food industry to reduce sodium in food and for health care professional organizations to educate Canadians and their membership about the risks. Specific actions with timelines for the government and the food sector were included in the policy statement [20]. A national media campaign was lead by the CSN to alert the public to the importance of dietary sodium reduction. A meeting of the food sector and the strategic planning committee organized by Dietitians of Canada resulted in an informal agreement to collaborate and to jointly request Federal Government oversight of a national process to reduce dietary sodium.

The NGO policy statement endorsed by 17 leading national health NGOs was publicly released at a national press conference organized by the CSN in October 2007. This was shortly followed by an announcement by the Canadian Minister of Health of the formation of an expert Sodium Working Group (SWG) to oversee the development of a national plan to reduce dietary sodium to recommended levels [21] as outlined in the policy statement. The SWG consisted of members from Health Canada, the Public Health Agency of Canada, the Canadian Institute for Health Research, Provincial and Territorial governments, the food industry and nongovernmental health and scientific organizations.

During this time research was conducted to examine and highlight the impact of high dietary sodium on hypertension and cardiovascular health and on the costs of health care in Canada. Notably a reduction in dietary sodium is estimated to reduce hypertension by about 30%, cardiovascular disease by 10% and direct health care costs of about 1.4 billion dollars a year [22,23].

A major program to educate the public and health care professionals on the health risks of high dietary sodium and how to reduce dietary sodium was the next step. Blood Pressure Canada developed a task force of 24 health care professional volunteers to assist the development and dissemination of information for the public, health care professionals and policy makers. The result was an extensive array of tools to educate the public and health care professionals about the health risks and how to reduce dietary sodium [20,24]. The Canadian Hypertension Education Program (CHEP), a national health care professional education program featured reducing dietary sodium as its theme in 2007 and 2008 and continues to highlight reducing dietary sodium as a major feature in hypertension education [25,26,27]. The CSN developed a website to consolidate information on sodium for the public and released guidelines on sodium levels in packaged foods [28]. Many of the national health NGOs that endorsed the policy statement have organized clinical and scientific sessions at regional and national meetings and/or published information for their membership on dietary sodium.

Perhaps most importantly the public has been informed through the significant media interest in the negative health impact of the high sodium levels present in processed, manufactured and restaurant foods. An active national media campaign in support of population wide sodium reduction has been sustained by the CSN and its NGO partners using earned media approaches such as awards and surveys. The media coverage has also maintained pressure on the Government and food industry to urgently address the sodium issue. A rough indicator of the extent of coverage was assessed using an advanced Google search (conducted 25 April 2011) with the essential words “Canada” and “dietary” linked to sodium or salt finds 8,320,000 “hits”.

## 3. Canadian Federal Government Activities to Reduce Dietary Sodium

In Canada several Federal Government organizations have played important roles in the effort to reduce dietary sodium. Health Canada is the Federal Government department responsible for food safety and some aspects of public health, the Public Health Agency of Canada (PHAC) is the Federal Government agency responsible for most public health issues, Statistics Canada is the Federal Government agency responsible for many health surveys and statistics and the Canadian Institute for Health Research (CIHR) is funded by the Federal Governmental to lead and coordinate health related research.

In 2004 Statistics Canada conducted a nutrition “24 h dietary recall” survey in Canada and in 2007, in response to Canadians interest in dietary sodium, analyzed the sodium intakes (Table 1). A subsequent media release from Statistics Canada focused on *the High Levels of Sodium Intake at All Ages* [14]. The survey results were used in extensive modeling exercises to determine the major dietary sources of sodium and how to reduce dietary sodium additives in foods to achieve various dietary sodium intake targets.

The collective efforts of the SWG lead by Health Canada resulted in the publication of the report “Sodium Reduction Strategy for Canada, Recommendations of the Sodium Working Group” in July 2010 [21]. In this report the SWG called for a reduction in population sodium intakes from the current 3400 mg/day to an average of 2300 mg/day by 2016, with an eventual goal of having most Canadians (95%) below 2300 mg/day. To meet the interim targets, the SWG called for a number of actions including (1) structured voluntary sodium reductions in the food supply; (2) public educational campaigns; (3) food science and health research related to sodium; and (4) planned, periodic monitoring and reporting of sodium intake levels and sodium program evaluation. This work has been extended by Health Canada’s publication early in 2011 of draft sodium reduction targets for a number of food product categories with targets set for 2016 and interim milestones for 2012 and 2014. These targets will provide a foundation for evaluating the success of the Canadian Sodium Reduction Strategy and determining if modifications are required in order to achieve maximum health benefits for all segments of the population. The Federal and Provincial Health Ministers also agreed that regulation to limit the sodium content of foods remains an option in case the voluntary approach lacks substantive and timely progress. On the eve of 2011, Prime Minister Harper indicated that the initiation of the sodium reduction program had been one of two health-related priorities in 2010 of the Federal Government, the other being child obesity. The CIHR hosted a meeting to define research needs related to dietary sodium followed by three priority calls with funding for sodium based research [29]; and also a media awareness meeting on dietary sodium. CIHR partially funds the Canadian Chair in Hypertension Prevention and Control. PHAC has conducted surveys of Canadian’s knowledge, attitudes and behaviours relating to dietary sodium [30] and funded a population survey of 24 h urinary excretion of sodium (results pending). In addition the PHAC and Statistics Canada in collaboration with NGOs have developed and conducted a national population survey of hypertensive Canadians that included attitudes and behaviours relating to dietary sodium (results pending). The PHAC has also contributed funding to the creation and dissemination of educational resources on dietary sodium to health care professional and the public through Blood Pressure Canada.

**Table 1:** Salt intake of Canadians from a 24 h dietary recall study *.

Age category	Gender	Average intake	% Above upper limit (UL)	Upper limit
1 to 3		1918	77.1	1500
4 to 8		2677	92.7	1900
9 to 13	Male	3513	96.9	2200
	Female	2959	83	
14 to 18	Male	4130	97.1	2300
	Female	2938	82	
19 to 30	Male	4066	98.8	2300
	Female	2793	76.3	
31 to 50	Male	3607	91.7	2300
	Female	2806	72.1	
51 to 70	Male	3334	85.7	2300
	Female	2537	62.3	
Over age 70	Male	2882	76.9	2300
	Female	2300	45.1	

Derived from Reference [14]. The survey does not account for salt added at home in cooking or at the table.

In Canada, provision of health services is a provincial responsibility and hence provinces are very interested in initiatives and interventions that would improve the health of their populations, reduce the incidence of chronic diseases, improve their management and decrease health care utilization. In 2010 the Federal/Provincial/Territorial (F/P/T) Ministers of Health supported the population goal recommended by the Sodium Working Group of 2300 mg/day as an average daily sodium consumption. There was also a commitment to develop a monitoring system for sodium levels in food to evaluate the effectiveness of voluntary industry measures.

Under the direction of the Deputy Ministers of Health, F/P/T senior officials have formed a Sodium Task Group to review the recommendations of the SWG. Beyond voluntary targets and monitoring, these elements include identification of potential federal and provincial/territorial regulatory mechanisms that may be necessary. A full report of the targets and timelines for foods, the monitoring and evaluation program, and the social marketing program is expected in the fall of 2011.

The SWG made many recommendations including consistency of serving sizes in Nutrition Fact Tables and onsite disclosure of nutrition information/menu labeling, food and nutrition policies for publicly funded institutions (e.g., schools and universities, daycares, health care facilities, recreation facilities), increased public awareness and education, reducing marketing of foods high in sodium to children, and research. Many of these elements are linked to or imbedded in broader healthy eating initiatives at the federal and provincial/territorial level and are already at varying levels of discussion and/or implementation across provinces and territories. To further support the effort a separate Federal Government committee (the Food Regulatory Advisory Committee) has also been charged with aiding the sodium reduction effort. The terms of reference for this committee with regard to sodium are currently being developed.

The Canadian government has also played a role in international efforts to reduce dietary sodium. Members from the Canadian government are members of the Pan American Health Organization (PAHO) Expert Group on Sodium Reduction, the government has helped support some of the PAHO Expert Group meetings and co-hosted one of the World Health Organization Platforms on Dietary sodium [31].

## 4. Food Sector

Although not outlined in detail in this review, the Canadian food sector has also played an import role in progress to reduce dietary sodium. About one-quarter of the membership of the Health Canada Sodium Work Group were from the food industry. The food sector including the restaurant industry has been negotiating reductions in sodium content of foods with the Federal Government and many companies have both made commitments and shown progress in reducing sodium levels in their food products. Some companies have also included public awareness of high sodium in foods as part of their marketing commitments.

## 5. Summary and Future Outlook

Canada has an evolving and already extensive effort to reduce dietary sodium levels towards those recommended by the Canadian Dietary Reference Intake values outlined in a report for the Institute of Medicine [15]. The program is currently lead by the Federal Government with substantial input from Provincial and Territorial governments and NGO’s. The Canadian program to reduce dietary sodium is maturing but can still be considered to be in early days. Education programs will need to be developed to be comprehensive and aimed all various sectors of the population including policy makers, the food industry, as well as the public. Greater analysis of the food survey will be necessary to determine vulnerable populations that may need specifically tailored knowledge translations interventions. NGO, governments, their agencies and industry will need strong commitment to achieve the 2016 target and ultimately a healthier and lower target for sodium intake. It is estimated there are over 50,000 processed foods that need serial reformulation over time in the effort to reduce dietary sodium. A critical aspect will be comprehensive monitoring and evaluation for the program to determine the successes and challenges and what additional revisions and steps are required. Although the sodium targets for food are currently “voluntary”, ancillary regulations relating food labeling, advertizing foods to children and package labeling would markedly aid the effort. Ultimately it is likely a regulatory approach to the sodium levels in foods will be required to ensure long-term healthy sodium intakes by Canadians. The initial voluntary approach allows time and flexibility for companies to reformulate foods given the complexity of Canadian’s food sources. But without all of the food processing industry voluntarily stepping up the plate, the sodium content in this large portion of the diet of Canadians will not be reduced enough for the target of 2300 to be met by 2016. Transparency and accountability from all partners will be required in this national public health effort. 

Further, consideration includes examining the impact of the salt reduction program on dietary iodine. Ongoing monitoring to ensure Canadians have adequate dietary iodine will be important as iodized table salt is currently used in Canada and reductions in salt consumption at home may cause iodine deficiency if there is inadequate evaluation tied to appropriate and timely intervention. Several international health groups have called for the use of iodized salt in all foods including commercially prepared foods or alternative sources of dietary iodine. 

Governments need to recognize that much of the chronic disease burden facing developed countries are caused in a large part by diets based in processed foods that have added sodium, simple sugars, saturated and trans fats. Processed foods need not be unhealthy. Regulatory approaches by governments have reduced the burden of diseases caused by micro-organisms and toxins in food and public water sources. This is perhaps largely due to the fact that micro-organisms and toxins are more directly understood to be linked to important diseases even in name (pathogens and toxins). It is time to call our diets pathogenic and harmful amounts of added nutrients pathogens. This change in nomenclature will aid the understanding that these foods and nutrients are not “treats” that are used for rewards or “junk” that is something useless we can do without but are major sources of premature death and disability.
